# Book Reviews

**Published:** 1813-08

**Authors:** 


### CRITICAL ANALYSIS
### *OF RECENT PUBLICATIONS*
IN THE
### DIFFERENT BRANCHES OF PHYSIC, SURGERY, AND MEDICAL PHILOSOPHY.

*A Treatise on the Diseases and Organic Lesions of the Heart and Great Vessels.* By J. N. Corvisart, M.D. &c. &c. &c. Translated from the French, by C. H. Hebb, Member of the Royal College of Surgeons in London. 8vo. pp. 404. Underwood and Blacks. 1813.

AS the French edition of this work cannot pass through many hands, we congratulate the reading part of the profession on the appearance of this translation, which, as far as we can judge, is faithful. The subject on which this volume treats is of deep importance, yet few writers have attempted it; hitherto, perhaps, the work of Senac may be regarded as the best, although many other authors have described affections of the organ; and recently an excellent treatise has been published by Mr. Burns, which, if not so complete as Corvisart's, contains much valuable information.

The present work is divided into five classes, which comprehend nearly all the diseases to which the heart, its coverings, and appendages, are liable.

The first class comprises the diseases *of the membranous coverings of the heart.*

The second, diseases *of its muscular substance.*

The third, diseases *of its fibrous or tendinous tissue.*

The fourth, of those diseases *which, at one and the same time, affect many tissues of the organ, and of those unnatural states which may be considered as so many organic lesions of the heart.*

The

The fifth treats *of aneurism of the aorta.*

In a preliminary discourse of considerable length, the author, with an animation, zeal, and eloquence, worthy of this subject, touches upon the necessity and advantages of the physician being minutely acquainted with anatomy—not from a barren curiosity to seek out singular appearances in the dead subject; not merely to inform himself of the names, form, and actual situation, of different organs; but to enable himself to distinguish diseases by certain signs, and not doubtful symptoms; always bearing in mind, that "the more minute anatomy is cultivated by physicians, the sooner will they be enabled by just observations to ascertain and prove the existence of a great number of organic lesions, of which most of them had not the slightest suspicion."

To the want of this knowledge combined with that of physiology, the learned author attributes much of that wavering, indecisive practice, and false diagnosis, which physicians often display at the bed-side; some accusing the liver, or the stomach, of derangement, when the complaint was in the chest; others mistaking a disease of the heart for asthma or dropsy; in short, no organ escaping their misapprehension.

But the physician must not rest content with mere anatomical knowledge, he must make himself master of the secret springs and working of the heart, and penetrate the veil which conceals the philosophy of mind. "Where, (says Corvisart,) may we exclaim, shall we find so clear-sighted a physician? No where, I readily admit; but I am not, on that account, the less fully convinced, that one of those qualities on which the *tact* of a great physician is firmly established, consists in that penetration, incessantly strengthened by exercise, which enables him to perceive in any given patient, the scene of his moral affections, while he observes all the physical phenomena which produce or which result from them."

While we admit the justice of these and other similar remarks of this author, for we cannot insert many of them, we may hint that they are perhaps more applicable to the French school of physic than to our own; though, in these our times, a great change has taken place in the education of physicians in France. Formerly, we find hardly a satirist or novelist who did not make them his especial butt; Boileau, Moliere, Rousseau, and a large fry of smaller writers, had exercised their wit so effectually on this unhappy tribe of men, that ridicule and physic were inseparable. In this country, however, satire has hitherto been very harmless, and has chiefly fallen upon some impudent pretender or hardened empiric. But let us not not boast, the French school is now a powerful rival to any that we can produce. Some of the best works upon anatomy, physiology, practical medicine, and chemistry, have issued from it. French physicians are emulous to cultivate general science, and are encouraged by honors, titles, and adequate remuneration for their labors, to assume that high rank in society which becomes their profession. The curious experimenter, besides the gratification of adding to the general stock of knowledge, obtains the notice of the world by his researches being recorded in the Memoirs of the Imperial Institute; chemistry opens a certain channel to rank and wealth; the great demand for skilful surgeons in an army constantly inspected by its active chief, insures their production; while peaceable men, disgusted with continued bloodshed and rapine, honor and esteem physicians whose labors tend to correct the evils and afflictions incident to human nature, and fearfully augmented by the ambition or the folly of rulers,

> "Quales ex humili magna ad fastigia rerum

> Extollit, quoties voluit fortuna jocari."

The first disease noticed by our author is Pericarditis, which he regards as an inflammation of the whole or of a part of the serous membrane which immediately invests and closely adheres to the heart in every part, and furnishes the loose bag in which it is contained. It appears in two forms, acute and chronic. Acute inflammation of the pericardium is not always easily distinguished. "Pleuripneumonia is generally complicated with it, and it is frequently very difficult to say which first appeared, or whether their commencement was not simultaneous." The author confesses he has not met with a pure case of acute pericarditis; of course, those which he cites are more or less complicated with other diseases; but as these appear to be chiefly pleuritis, peripneumonia, and paraphrenitis, it is of less consequence, as the only hope of cure in either of them, pure or combined, must rest on early and copious bleeding.

The author has described a variety of the disease under the term sub-pericarditis, a case of which we here insert.

"On the 9th of January, 1799, a man, aged forty years, received a blow by the fist on the region of the heart. On the 25th of the same month, violent febrile symptoms, accompanied by oppression and pain under the left part of the sternum, suddenly took place: during the three first days these symptoms increased so much, that on the 30th he made up his mind to become a patient in the Clinical Ward of the Hospital; the best marked inflammatory symptoms had then disappeared without any visible advantage: he complained only of a slight pain in the head, and a continual and indescribable anxiety, which which left him not a moment's ease; the skin was dry and hot, the pulse small, frequent, unequal, intermittent, and irregular; the eyes were sunk; the features much changed; the left cheek was very red; the mouth tolerably clean. The sound, by percussion, was very obscure throughout the left side. The breathing, apparently easy, was, nevertheless, on close observation, short, frequent, and a little interrupted; the cough dry and not painful; the patient, notwithstanding, complained of a pain which extended from the posterior part of the sternum to the left side, and to the lower part of the right side of the chest; there were momentary sinkings, which did not amount to fainting. The stools were few, the urine was turbid and deposited a sediment. On the first day I ordered one bleeding; but I did not persevere in this plan, on account of the advanced stage of the disease. From the 30th of January, it was evident that the disease made a rapid progress; the countenance became more and more *hippocratic*; the patient had not a moment's quiet; the breathing was continually interrupted and very difficult; the pulse vacillating, and scarcely perceptible; the prostration of strength extreme, notwithstanding the use of cordials. He remained in this state for the ten first days he was in the hospital; the only remarkable phenomenon during that time, was a spontaneous and almost sudden dissolution of the right eye, from a suppuration which took place in it without being preceded or attended by any inflammatory symptoms. After these ten days, the disease appeared still to advance more rapidly. The features became entirely changed, the pulse imperceptible, the debility extreme, even unto fainting. This patient finally sunk on the nineteenth day of his admission into the hospital, and on the twenty-fourth of his disease.

"I sought in the cranium the cause of the sudden destruction of the right eye; but the brain, the thalami nervorum opticorum, and the optic nerves themselves were perfectly sound.

"The pericardium was so enormously enlarged as to contain near four pints of seropurulent fluid; its inner surface was throughout covered with a thick crust of albuminous matter, the superficies of which was reticulated and *curdled;* an appearance of which one cannot give a better idea, than by comparing it to the inner surface of the bonnet, or second stomach of a calf, except that, in this case, the depth of this kind of net-work was not so great.

"The heart was not altered in size; but the leaf of the pericardium which covers it, was firmer and more than two lines thick. The fleshy fibres were not sensibly altered. The left lung, pushed upwards, was spongy and crepitous; the right lung was sound.

"That part of the diaphragm which unites with the pericardium was not inflamed."

The author attributes the freedom from complication in this case, to the exciting cause being external and circumscribed. He also thinks that if antiphlogistic means had been actively employed in the beginning, they might have moderated, and perhaps subdued, the inflammation.

The chronic form of pericarditis is complicated and obscure, scure, and Corvisart has afforded us no additional knowledge upon the subject. The treatment he recommends in all forms of the complaint, is such as is most generally pursued by sensible practitioners.

*Adhesion of the pericardium to the heart*, is a frequent consequence of pericarditis; from whatever cause it is produced, Corvisart states that it may take place in three ways. 1. From interposition of the lymphatic exudation thrown out by the inflamed pericardium. 2d. From rheumatic and gouty affections. 3d. " It is sometimes owing to numerous cellular filaments, the length of which varies from seven or eight lines to the smallest possible." " The adhesion of the pericardium to the heart, in this last case, and sometimes in the second, does not constitute a real disease; it merely puts the patient into a state of insupportable inconvenience." In partial adhesion the function of the heart is not sensibly injured ; but when the adhesion is complete, the author thinks that death must inevitably take place, though not immediately. The following is a case in point:

" A man, forty years of age, felt a very violent pain in the epigastric region. This pain was accompanied by weak but frequent palpitations, and great difficulty in breathing. The pulse was small, quick, and irregular; and on applying the hand to the region of the heart, it appeared to beat with irregularity. From time to time the pain, the difficulty of breathing, the palpitations, and indeed all the symptoms, were greatly aggravated. In one of these paroxysms, which occurred after short intervals, an ecchymosis took place about the eye-lids of the right eye, the ball of which became inflamed.

" Although the number and severity of these symptoms gave great reason to fear for the life of the patient, he was, nevertheless, enabled by the antiphlogistic treatment, and the use of emollients and antispasmodics, to resume his usual occupations. He continued in health for forty days, when he became a patient in the Clinical Ward. To the symptoms before described, were now added ascites, and frequent paroxysms of fever. The quantity of water was so great as to render tapping necessary, but this was employed only as a palliative ; in effect, the water again collected; the patient complained of continual pains in various parts of the belly, but principally at the bottom of the right iliac region. The pulse was constantly very weak, the sleep trifling, and the left side of the chest did not sound on percussion. The strength diminished daily, notwithstanding the use of cordials. In fine, eight months after the first illness which I have mentioned, the patient passed quietly from life to death, a few minutes after he had gone to bed, where he was thought to be sleeping.

" In the course of this person's first complaint, I had announced the existence of an organic lesion of the heart. My diagnostic became more precise long before his death, and I thought I could pronounce that there was an adhesion of the pericardium to that viscus. On opening the body I found a considerable quantity of water in the left left cavity of the chest; the pericardium adhered externally to the lungs, and internally to the heart, its adhesion to which was so strong, that they could not be separated without a most careful dissection. The blood was accumulated in the right cavities of the heart and in the venæ cavæ, so as to distend them to an extraordinary size; the other parts of this organ were in a natural state. The blood, in all these cavities, had a remarkable fluidity. The muscular fibres of the heart were generally very pale, and their action must have been reduced to almost nothing for some time before death, which, perhaps, may have been the immediate cause of that event. The left lung, pushed up towards the superior part of the chest, was hardened; the right was in a tolerably good state.

"The cavity of the abdomen contained a good deal of bloody serum; the alimentary canal was contracted, and altered externally; almost the whole of the peritoneum was covered with granulations."

Besides the symptoms already detailed, in some cases of adhesion of the pericardium others have been frequently observed. In many instances, sudden flushing of the countenance; a painful sensation of pulling in the region of the heart; the breathing quick, and oppressed on the slightest motion, and faintings; with a pulse more or less irregular. In distinguishing this complaint from other affections of the organ, the author places great confidence in the absence of palpitation; for how, he asks, can the heart, when closely connected with the diaphragm by its membranous covering, perform violent and tumultuous movement, all change of position being nearly prevented by the adhesion? "The contractions of the heart are, in this case, quick and disordered, but dull and deep, weak, obscure, and imperfect." The complaint, however, is always difficult to be ascertained, especially when combined with other affections of the heart or chest; and complete adhesion has been discovered by dissection, when during life such an affection was not indicated by a single symptom.

Among several cases cited by the author to illustrate this disease, is one of an apothecary's assistant, who had long appeared dejected, and at length committed suicide. In this case the pericardium had formed adhesions for the space of about two inches in diameter round the apex of the heart, which appeared of long standing.

Chapter II. on Hydro-pericardium, though not devoid of interest, does not claim our peculiar attention.

In the Second Class, which treats of diseases of the muscular substance of the heart, Aneurism, which is here synonymous with dilatation of the heart, holds a conspicuous rank, and occupies much space; we shall however pass it over in our analysis, because, from the labors of our own surgeons and anatomists, we are perhaps better acquainted with this than than some other diseases of the organ which we shall have occasion to notice.

In the fourth chapter, upon *Induration of the muscular Tissue of the Heart*, some interesting cases are detailed. We extract one from section 2. " Of the Conversion of the muscular Tissue of the Heart into a cartilaginous or osseous Substance."

" A man, aged 64 years, had always enjoyed good health until the beginning of the summer of 1798. At this time he was attacked with a disease of the chest, for which he was bled seven times, and which, on his recovery, left him in a state of the greatest debility.

" Fourteen months after the cure of this acute disease, he was seized with difficulty of breathing, with violent stiflings, which had existed for about two months when he came into the Hospital. Six days before his entrance, the stiflings had become so violent, that, on the slightest motion, he was in danger of suffocation. He said he had not had palpitations. His pulse, scarcely perceptible in either wrist, was small, concentrated, and irregularly intermittent; it might be said to be suspended for two or three pulsations: on placing the hand on the region of the heart, it was felt to beat with great violence, which by no means corresponded with the state of the pulse. The chest sounded well, except in this region and towards the posterior and right side of that cavity, which had been the seat of the peripneumony. The legs were rather clammy than œdematous. He often awoke from sleep with starting.

" Though convinced of the existence of an organic lesion of the heart, I felt much difficulty in deciding upon its nature. I was most disposed to consider it as an aneurism of that organ, complicated with the contraction of some aperture.

" The prognostic appeared to me of the most melancholy kind. However, diuretics procured sensible relief for two months. The breathing became easier, the pulse more free and regular, and less intermittent; but towards the beginning of April, 1800, he became worse than ever. The pulse resumed its old characters. The breathing became suffocating, and the urine less in quantity. The legs again began to swell. The œdema made an alarming progress in a short time, and the patient died without agony on the 17th of April, eighteen months after the disease of the chest, and two months after his admission into the Hospital.

" At the time of opening the body, the face, and particularly the lips, were of a violet and blackish color.

" The lungs, in other respects sound, had contracted slight adhesions to the costal pleura.

" The size of the heart was much greater than usual.

" The right auricle and ventricle were much expanded, and their communicating aperture was evidently in a state of dilatation. The left auricle, too, was very large, and its aperture of communication dilated; the mitral valves were become cartilaginous.

" The sides of the left ventricle were at least an inch thick, and very solid; the apex of the heart to a certain height and throughout its substance, was cartilaginous; the columnæ carneæ of this ventricle had also acquired a remarkable hardness approaching to that of cartilage, of which they had the physical characters."

In the fifth section the author treats of the conversion of the muscular tissue of the heart into a fatty substance. Haller, and Vicq. d'Azyr, with many other writers, have described the change of the muscles into a fatty substance, and certain modern anatomists have averred the same of the heart; but Corvisart, without denying the possibility of the fact, declares that he has not met with such an occurrence. Our readers will be aware that he does not mean those cases in which the heart is encumbered and loaded with fat, which is not an uncommon event.

The chapter on the induration or ossification of fibrous parts, contains much interesting matter. As a specimen we select a case of *Contraction of the left Auriculo-ventricular Aperture.*

"A blacksmith, aged 20 years, of a very robust constitution and of a sanguineous temperament, came into the Hospital of la Charité on the 4th of June, 1792. He came there, he said, on account of a dysentery with which he had been attacked in the winter, and which had caused him to lose much blood by stool. He never had had any other complaint, but he was subject to nasal hæmorrhages, to which he observed all his family were liable.

"For the last ten or eleven months he had been unable to take any violent exercise without experiencing some pain in the chest, and strong pulsations in the region of the heart. Tne nasal hæmorrhages had ceased for three months before he came into the Hospital. The palpitations, then more frequent, became also more violent, but still without being excessively distressing. He came to the Hospital for his pretended dysentery, and did not at all complain of the above-mentioned symptoms, which he only acknowledged on being interrogated.

"As soon as I saw the patient, I suspected an organic lesion of the heart, and two days after I announced its existence in one of my private clinical conferences. Here follows what was most remarkable.

"On placing the hand on the region of the heart, strong, quick, and very irregular palpitations were felt. The patient could not lie on his back, in consequence of its producing a sense of threatening suffocation: he lay tolerably comfortably on the left side, but often awoke with startings, and said that he felt, while sleeping, violent blows on his body. The pulse was irregular, frequent, hard, weak, and rebounding: it had very irregular intermissions, and was altogether so variable, that it is difficult to convey a correct idea of it.

"This disease once recognized, the intensity of the symptoms readily enabled me to point out the prognostic: it was inevitable death. I omit giving a detailed account of the treatment, which consisted sisted in small bleedings, mild diuretics, antispasmodics, and other palliatives, the inutility of which I had before pronounced.

" The symptoms soon made a very rapid progress, and the patient was convinced, from his own feelings, that he carried in his breast the cause of death. The sense of suffocation, which had existed for some time, became more and more alarming, the lower extremities began to swell considerably ; a violent delirium came on, which lasted for near twenty-four hours ; an excessive coldness of the limbs took place, and the patient died on the 29th of June, twenty-five days after his admission.

" The body was generally œdematous. There was a slight effusion in the cavities of the chest. The lungs were sound; the pericardium contained a little water. The heart was very voluminous, and all its cavities were gorged with blood.

" The right auricle and ventricle were in a natural state, except that they were enlarged, as was their aperture of communication. The left auricle was also dilated: its opening into the left ventricle was exceedingly contracted, and formed a kind of osseous slit, through which a thin coin could scarcely have passed ; the part of the mitral valve which adapts itself to the orifice of the aorta, fitted but very irregularly.

" The large vessels were in a natural state.

" The organic derangement which I have described, was evidently the cause of the increased volume of the heart, and of death.

" It is clear that the disease of which the patient complained was not dysentery, but only an effect of sanguineous plethora, determined more particularly to the venous system of the lower belly. This may easily be explained ; the left cavities of the heart, not properly emptying themselves, the right could not become unloaded ; the blood, therefore, from the venæ cavæ accumulated ; and hence arose the sanguineous distention of the liver, so frequent in diseases of the heart, as also the hypogastric venous plethora, and the intestinal hæmorrhage, but not *dysentery.*

" The temperament of the patient was sanguineous to an excess; it was that of all his family ; he was subject to nasal hæmorrhage ; ten or eleven months before his death, he could not move without bringing on palpitations. The bleeding at the nose ceased, the palpitations increased, and the intestinal hæmorrhage came on. Is any thing more necessary to prove, that this disease was not *dysentery?* It was also unaccompanied by intestinal pains, tenesmus, or any other symptoms of this affection."

Contraction of the right auriculo-ventricular aperture from cartilaginous or osseous induration, is rare. In the case stated by Corvisart of a man aged 60, the chief symptoms were bloatedness and violet hue of the face, lips, and neck ; extreme difficulty of breathing ; beating of the heart diffused and irregular ; the pulse irregular, and not isochronous with the pulsation of the heart ; confusion of ideas followed by drowsiness succeeded ; the pulse sunk.

At the time of opening the body, the face was violet and blackish ; blackish; the lungs adhered on every side to the internal surface of the parietes of the chest. The right auricle was much enlarged; the tricuspid and mitral valves were cartilaginous, which diminished the diameter of each orifice; the pericardium contained some water; the dilated aorta had some ossified points on its internal membrane.

Two sections follow upon the cartilaginous or osseous induration of the auriculo-ventricular valves; and of the semilunar and sigmoid valves; lesions to which the heart is very subject, and which may partially exist without much inconvenience being experienced. They sometimes, however, prove fatal.

"A washerwoman, aged 76 years, was admitted on the 14th of May, 1803, into the Clinical Ward. This woman had never had good health; at the age of 67 years, she experienced so much difficulty in walking, that she was often obliged suddenly to stop. This first symptom was accompanied by palpitations. Her complaint did not become alarming for eighteen months after. At this time her extremities were so anasarcous, that she was obliged to give up work.

"When she came into the hospital, her countenance was livid, her eyes were watery; the lower extremities, the arms, the hands, and the parietes of the abdomen, anasarcous. She had frequent nausea; her breathing was high, short, and interrupted; the palpitations were frequent; the chest, when struck, did not sound in the region of the heart; the pulse was quick, rather weak, and irregular. These symptoms were sufficient to point out the organ affected, and the danger of the disease.

"Thirteen days after her entrance into the hospital, this woman, whose disease during that time had made a rapid progress, died as if suffocated.

"The face, in this subject, was unequally black and livid; the brain in a healthy state; the lungs were flabby and anasarcous; and there was a small quantity of water in the two pleuras. The pericardium contained about half a pint of serum; the heart was not much enlarged beyond its natural size.

"The right ventricle was soft and flabby to the touch, as it usually is. The left, on the contrary, resisted pressure, with so much force and elasticity, that its parietes immediately returned to the state in which they were before they were compressed. The substance of this ventricle was so firm, that it preserved almost a cylindrical shape. A portion of this cylinder was covered by the pericardium; and the other part, forming the septum, projected into the right ventricle, the cavity of which it in a great degree occupied. Its fleshy parietes were fifteen lines thick.

"The left ventricular aperture was studded with many sharp and osseous points, which, uniting near the septum, formed a substance of considerable size. The mitral valves were indurated only where they came into contact with the partly osseous circle of the ventricular orifice.

orifice. The mouth of the aorta did not appear contracted, but the semi-lunar valves, by their arrangement, nearly closed up its area.

"These valves were not only indurated and ossified, but much thickened by the calcareous matter which was deposited between the two membranous folds which form them. Their osseous hardness kept them in a sunk and immoveable state. Their free edges approached so as to touch each other, and almost entirely to obliterate the area of the vessel. Had not the base of one of these valves, although ossified and thickened, preserved sufficient pliability to perform a kind of see-saw motion, which augmented, by a line or two at most, the opening for the passage of the blood, it would have had to pass out of this ventricle by an excessively narrow slit.

"The right ventricle was in a natural state. The auricles were not enlarged, but their parietes were so weak that in many places they were transparent, and tore, with the greatest ease, on separating them circularly from the base of the ventricle. All the cavities of the heart were distended with black blood, partly fluid and partly coagulated."

The chapter on Excrescences of the Auriculo-ventricular and Semilunar Valves, contains some interesting cases, and practical observations.

The Fourth Class of our author's arrangement comprehends *Diseases which affect at the same time several Tissues of the Heart.* Carditis is the head of the first section. This formidable disease, it seems, is less known than its importance would lead us to suppose: in many instances it exists where not suspected, and Corvisart has therefore divided it into *occult*, and *manifest*. The cases of the former adduced by the author are too valuable to be omitted, although our extracts have already been numerous.

"A cleaner of shoes, aged 67 years, of a sanguineous temperament, had for thirty years been subject to a distressing dyspnœa, and frequent colds, but had never felt palpitations.

"On the 24th of April, 1803, the breathing being more than usually difficult, he felt a slight pain in the middle and interior part of the left side of the chest; two days after he spat blood, and on the 29th was admitted into the Clinical Ward. He had then a little pain in his head; his countenance was animated; his eyes brilliant; his tongue whitish; the breathing a little difficult, with wandering pains in the chest, but no palpitations. His pulse was weak, irregular, intermittent, and not the same in each wrist. On the following day, the respiration was more difficult, noisy, and rattling; the pain in the chest had greatly increased; he had delirium, and talked much. In the morning he got up several times to the window to breathe; he did the same at mid-day, lay down and died unexpectedly on the 7th day of the disease.

"The pain in the chest, the difficulty in breathing, and the external examination, had, from the second day, convinced me of the existence of peripneumony; the brilliant eye, the talkativeness, the seat of the

 pain, pain, and the characters of the pulse, indicated to me that the inflammation had extended to the heart, without leading me to suspect any particular derangement in the texture of that organ.

"At the time of opening the body, the countenance was unequally livid and violet, and the subcutaneous veins was gorged with blood. The brain was in a good state.

"The right lung, throughout adherent to the pleura, was flabby, very extensible and œdematous. The left lung was covered throughout a great part of its surface, particularly near the pericardium, with a pseudo-membranous crust several lines thick. The costal pleura of this side appeared to have been the seat of inflammation.

"The pericardium contained about a pint of a purulent and flaky fluid; its internal surface was covered with a membranous-like substance, the superficies of which had numerous elevated points upon it.

"The heart, of the natural size, was soft and flabby. The fleshy parietes of the ventricles and auricles were of a pale yellow; a fatty substance might be said to be deposited between their fleshy fibres, which seemed separated from each other. A vascular kind of network was perceptible upon the surface, and even on the interior substance of the fleshy parietes of the heart. By gently pressing the fleshy substance between the fingers, it was easily reduced to the consistence of a pap, and of a dull pale color. All the cavities of the heart contained polypiform concretions, which extended even into the cavities of the vessels.

"The abdominal viscera were all sound.

"To this fact, and which I will call obscure acute carditis, I will add two others perfectly analogous, and in which the diagnostic was as obscure as the pathological state was evident on opening the body.

"A young person came into the Clinical Ward, laboring under a disease of the chest, with a pale and wan countenance, and œdematous legs; she coughed much, and had a puriform expectoration. Finding little relief from the medicines which she took, she quitted the hospital.

"She soon returned, and the same means were again employed, as I still entertained the idea that it was a pulmonary affection: but, on a more careful examination, I observed that she had constantly a small pulse; and the lips being bloated and violet, a symptom not common in pure phthisis, I used percussion with more care, and found that the chest, which sounded well on the right side, gave no sound on the left. This, and several other symptoms, made me think that the left cavity was full of liquid. The belly was so distended with water, that I ordered paracentesis, which gave vent to a considerable quantity; but the patient died in a few days.

"On opening the body, the pericardium was distended with a purulent fluid. The surface of the heart was covered with a layer of lymphatic matter; the heart itself was small, contracted, soft and pale, even in the interior of its substance. The interior surface of the pleura was only affected on the right side."

"A woman was received into the Clinical Ward. She had ascites, for which she received the usual treatment; was tapped several times, without finding more relief than is usually experienced in in similar cases. Although the symptoms were exceedingly obscure, I had an idea that they might arise from an affection of the heart. The smallness, weakness, irregularity, and peculiarity of the pulse, gave rise to this idea. This patient died; and I found, on opening the body, the pericardium much distended with a lactescent and puriform liquid. The heart was whitish, small, as if retracted, without consistence, and quite misshapen. The auricles, ventricles, and great vessels, were surrounded by a white lymphatic substance.

"This woman had been ill for seven or eight months; but the true nature of her complaint had never been suspected: yet the appearances, on opening the body, left no doubt of the early existence of carditis, which had degenerated into chronic inflammation of the heart, the symptoms of which were obscured by the dropsy, consecutive, doubtless, upon the affection of that organ."

Acute carditis, according to Corvisart's own experience, never occurs without complication; but he does not appear to have seen many instances of the disease. It may terminate by suppuration, by gangrene, and by ulcers.

The second section treats of *Rupture of the Heart.* This accident may be either complete or partial: in the one case, the parietes from the interior to the exterior surface burst, and the blood is effused into the cavity of the pericardium; in the other, only a portion of the substance of the heart is affected. Complete rupture of the heart is a rare event: the ingenious translator has reminded us that George the Second died of this complaint, and has given Dr. Smollett's concise account of the circumstance. Under partial rupture of the heart, the author includes the rupture of one of the carneæ columnæ, on the internal surface of the ventricles; and "the rupture of the chordæ tendineæ, which, rising from those pillars, are attached to the floating extremities of the valves." This affection has been noticed, and the probability of its occurrence testified by Haller, Morgagni, Senac, and other writers; but very few cases of it have been recorded, and none so distinctly as those related by Corvisart.

"A man, aged 20 years, of a vigorous constitution, was admitted into the Hospital of la Charité, at an early period of the French revolution. For some time he had quitted a sedentary trade to become a messenger: devoted to this very laborious life, he travelled incessantly to every court in Europe. When he came into the hospital, he had been travelling a thousand leagues on horseback, without taking repose: he had, besides, taken a journey from London to Paris; and, in passing from Dover to Calais, he had experienced, for the first time, difficulty in breathing, and a spitting of blood; having, notwithstanding these symptoms, continued his journey, the disease had much increased, and by the time he had reached Paris, was accompanied by pain in the chest. He had been bled five times during the three days he had kept at home, but finding no relief from the bleedings and other treatment which had been deemed necessary, he came to the hospital eight days after the commencement of the disease.

"The features of the face were then much altered, and the extremities slightly œdematous; the pulse was small, hard, very frequent, and rather irregular. On applying the hand to the region of the heart, besides the very strong pulsations of that organ, confused and irregular beatings were felt, not at all resembling its natural movements. The patient could not continue long lying, standing, or sitting, and was in a state of anxiety and agitation not to be described.

"His legs and thighs swelled the day after his entrance, and his features became still more changed. During the following night, the symptoms increased: he was dreadfully agitated, walking into the other wards, sitting down, incessantly rising, but still preserving his mental faculties. The sense of suffocation now becoming highly alarming, this unfortunate man was convinced of the danger of his situation, and abandoned himself to the most violent despair: he died, testifying by all his gestures the regret he felt at quitting life.

"I repeated, before I opened the body, what I had announced on the first day, that there existed in this patient an acute lesion of the heart, and probably a rupture or laceration of some part of it.

"The left lung was sound; the right had contracted slight adhesions to the costal pleura; its upper lobe was very firm, but without tubercles; in the ridges which separate the different lobes, there was a lymphatic coat, produced by the consecutive inflammation of which this organ had evidently been the seat. There was a quantity of water in the chest.

"The pericardium contained about half a pint of yellowish serum. The heart had not acquired an extraordinary size. One of the large columnæ which support the mitral valves of the left ventricle, had ruptured at its base, so that it floated loosely in that cavity; there was an appearance of suppuration at that point of the heart where the rupture had occurred, proving that it was not of long standing. Near the rent there was a clot of blood covered with purulent matter, which came from the torn part."

"A turner, aged 34 years, of a strong constitution, and of an irritable and violent temper, gave himself, in endeavouring to move a tun of brandy, to use his own words, a *twist of the reins*, which immediately caused a sense of suffocation and an acute pain between the shoulders: soon afterwards, cough, palpitations of the heart, and frequent startings in his sleep, took place. These alarming symptoms, far from yielding to the remedies used, and becoming every day worse, he went to the Hospital of la Charité, where, during his first residence, he received much relief. He quitted it, but returned in four months, when nearly two years had elapsed since the violent exertion which appeared to have produced the disease.

"On the 24th of March, 1803, the day on which he came into the Clinical Ward, this patient was in a state of extreme anxiety; his breathing was suffocative; and he felt acute pains in the region of the heart, which forced him to cry out, particularly during the night. He died in two days.

"On opening the body, I found, in the right cavity of the chest, four four pints of serum. The lungs of this side were sound. The left thoracic cavity contained but little water.

"On the fore part of the mediastinum there was a small purulent spot of the size of a sixpence, without any alteration of the cartilage of that side, to which this kind of ulceration corresponded. The heart, with its capsule, was three times its natural size.

"On the exterior surface of the pericardium, there were a number of excrescences, shaped like a cock's-comb, pale and livid at the base, but of a deep red at their summit: they were nothing but portions of fat which had undergone a peculiar kind of change. This membranous capsule adhered to the heart by means of a firm fatty substance.

"All the cavities of the heart were dilated, and contained much black and coagulated blood.

"The right auricle was so much dilated, that its auricular appendix had entirely disappeared; the right auriculo-ventricular aperture was also much dilated, and the ventricle slightly so; the tricuspid valves, and those of the pulmonary artery, were in a good state.

"The left auricle and ventricle had much increased in volume. The parietes of the ventricle were thickened, and the mitral valves studded with several soft and fleshy-like excrescences.

"On examining the tendons of the fleshy pillars which support these valves, two of them appeared to have been ruptured for some time. Their extremities were soft, smooth, and rounded at the ruptured part. The precise point of the edge of the valves to which they had been attached, could not be discerned.

"The valves of the aorta were not altered, but this artery was itself dilated at the beginning of its arch.

"There was some water in the abdomen; the viscera of this cavity were sound."

The third Section, upon *Tumors of the Heart and other unnatural States of that Organ*, contains some valuable and interesting facts both from the author's own experience and that of other writers. The following case of *Perforation of the Septum of the Ventricles* is very curious, and deserving of attention.

"A child, aged 12 years and an half, was admitted, on the 21st of April, 1797, into the Clinical Ward. He was then in so dreadful a state, that there was reason to think he could not live many days. His complaint, according to his own account, had not existed for more than five months; but, from the violent and repeated palpitations which he had always experienced, it was not unreasonable to suppose, that the disease was of much longer date.

"When this boy was admitted into the Hospital, his face was bloated and his lips were violet; there was no œdema in either the upper or lower extremities; the respiration was much impeded; the hand, placed upon the region of the heart, felt an irregular beating, accompanied by a very remarkable rustling. The pulse was, nevertheless, very regular, but small, weak, and easily suppressed, The palpitations were frequent, and came by paroxysms, accompanied by

 threatening threatening suffocation. The patient could not lie down: he was better when sitting upright, and still more so when inclining forward. He made water frequently, and in proper quantity.

"During his short stay at the hospital, his disease made an alarming progress. He was put under a course of diuretics and powerful antispasmodics, but they procured, as had been foreseen, no relief.

"On the 24th he had a fit of suffocation, stronger than any which had preceded it, and which threatened to terminate his existence; but this paroxysm went off, and the patient, some moments after, found himself better than he had been for a long time. However, more alarming symptoms soon returned, and he died on the 25th, after an agony of from ten to twelve hours, during which his body was covered with a cold sweat, and a yellowish froth dribbled from his mouth,

"On opening his body, the lungs, except being rather flabby, were in a healthy state. The pericardium contained a small quantity of water.

"The heart was much enlarged, and appeared rather to belong to a large and full-grown man, than to so young a child. There was nothing remarkable in the auricles but their increased size.

"The parietes of the right ventricle were thicker than usual. The septum had preserved its natural thickness, but there was a round hole in it, near the spot where the pulmonary artery takes its rise, large enough to admit the extremity of the little finger. This opening communicated with the cavity of the left ventricle; its edges were smooth and whitish throughout. At the upper part of the margin of this aperture, there were two little fleshy tubercles, of a reddish color.

"The parietes of the left ventricle had preserved their natural thickness. In the cavity of this ventricle, just below one of the sigmoid valves of the aorta, the opening of the hole I have been speaking of, could be perceived. The aortic semi-lunar valve, beneath which it was situated, was corroded, and in part destroyed. It formed a kind of little fringe, which presented itself at the orifice of communication without entirely closing it; so that the blood, propelled from the left ventricle into the aorta, could, when this ventricle ceased to act, flow back, on account of the destruction of the sigmoid valve, into the right ventricle, by passing through this unnatural opening, the direction of which, however, appeared to be from the right towards the left ventricle.

"An important question, which, after the details of the case, and the examination of the body, seems still undecided, is, whether this opening existed from birth, or were accidentally formed by rupture or erosion. In the first case, it might be considered as a vicious conformation; in the second, it should be placed in the class of organic diseases. The smooth and tendinous-like nature of its edges favor the former opinion, while the erosion of one of the semi-lunar valves extending even to the margin of the opening, and the existence of the tubercles, incline one to embrace the latter."

Having far exceeded our usual limits, we must refer to the book itself for the fifth class, which treats of *Aneurism of the Aorta*, assuring our readers, however, that they will not be disappointed if they expect to find much useful information upon the subject. The volume terminates with some able corollaries from the diseases of which it treats.

On the whole, we have been highly gratified by the perusal of this work, which ought to be studied by all practitioners, for we think all might derive benefit from it. The translator appears (for we have not the original by us) to have executed his performance with care; and the language, though sufficiently grave for the English, is not devoid of the vivacity of the French idiom.

---

*Some Account of Mary Thomas, of Tnnyralt, in Merionethshire, who has existed many years without taking Food; and of Ann Moore, commonly called the Fasting Woman of Tutbury. Accompanied with Portraits and illustrative Etchings. By* James Ward, Esq. R. A. Imp. folio; pp. 11, and seven plates; Lond. 1813.

The case of Ann Moore of Tutbury, the woman who assumed the privilege of living without food, has been so much before the public, is so very generally known, and its fallacy has recently been so fully exposed, that we might be excused filling our pages with any further details on this subject, did not this elegant work, by Mr. Ward, demand from us an attention not due to the numerous herd of ephemeral pamphlets.

Mr. James Ward, an artist of great talent and deserved celebrity, having been called, in the course of his professional pursuits, into the neighbourhood of Ann Moore, made her several visits, and became convinced of the honesty and truth of her assertions. Impressed with this feeling, and looking upon her to be an extraordinary instance, perhaps, of a direct visitation from the Supreme Being, altering the accustomed course of the laws of Nature, for some inscrutable purpose, he investigated her history with minuteness, and examined her form and general external appearances with the eye of an artist. The result was the detail now before us, accompanied with two etched portraits, the value of which will probably deliver her history down to posterity, and record her imposition to future generations.

About the period of Mr. Ward's visits to Ann Moore, accident brought him acquainted with Mary Thomas, a poor Welchwoman of Merionethshire, whose history, resembling in some points that of Ann Moore, was still more extraordinary, because her morbid state was much severer, and had

 been been of longer duration, comprehending the greater portion of a century. The sketch he gives of this woman's strange history is accompanied also with etched portraits and engravings, illustrative of various interesting localities connected with that history.

From this narrative it appears, "that Mary Thomas has existed between seventy and eighty years, almost without food; and certainly, according to evidence that does not appear in any way objectionable, for ten whole years, without the least particle of nutriment of any kind or form passing her lips, and without showing any sensibility or knowledge of external events; and has had, in that time, no excrementitious discharges from the intestines or urinary bladder. In 1812 this woman was still living; and, from the extraordinary tenacity to life which she evidently possesses, under circumstances that would have abridged the days of any other human creature, though now 80 years old, she may, perhaps, long enough survive to have her history more explicitly detailed, and the facts connected with her peculiar state decidedly unfolded."

This expectation has, however, been defeated by the death of Mary Thomas this year.

On inquiring into the history of Mary Thomas, a fact has arisen of some importance. Mr. Pennant, whose reputation for every thing excellent is still fresh in our minds, saw Mary Thomas in the year 1770; and his relation agrees so much with Mr. Ward's, that they mutually support each other, and give a degree of credibility to an otherwise incredible case.

The extreme emaciation of Mary Thomas is happily shown in the etchings; and indeed nothing short of these *fac-similies*, as we are assured on the best authority they are, could distinctly point out the absolute loss of muscular fibre, and the diaphanous representation of a skeleton dimly seen through the shrivelled integument.

Prior to the public disclosure of Ann Moore's imposition, Mr. Ward seems to have entertained some degree of doubt respecting her veracity, as we judge from the concluding sentence of his work.

"But in whatever degree (he says) the case of Ann Moore may be equivocal, the circumstances which have given rise to that equivocability do not apply to the case of Mary Thomas. She is emaciated, poor, friendless, and almost unknown. No licentiousness has disgraced her early life; no charge of former imposture is alleged against her; there are no objectors to her veracity; and her abstinence continues after the impressions excited by its novelty have long worn away. Forty years ago she was seen and examined by a gentleman fully fully competent to judge of her case, and he does not hint a doubt of its truth. When Mr. Pennant visited her in 1770, he found the neighbourhood convinced of her long and preternatural fasting: no suspicion had ever arisen of any fraud being practised; no motive for imposition offered: few persons saw her, the neighbors excepted, but those who, like Mr. Pennant, were seeking to elucidate the natural history of the county, or to develope its picturesque beauties."

Reluctant as we are to admit any of these extraordinary cases, as plain matters of fact, both on the ground of principle and on history, we cannot but allow a degree of credit to Mr. Ward's "unvarnished" account of Mary Thomas; and regret, with him, that her death should have prevented a minute investigation. But, whatever our opinions may be, we consider this to be a valuable document, as recording extraordinary aberrations of either body or mind; and as accompanied with etchings of the highest excellence, we unreservedly recommend it.

---

*Medico-Chirurgical Transactions.* Published by the Medical and Chirurgical Society of London. Vol. III. 8vo. Lond. 1812.

The rapid publication of the Transactions of this respectable Society, shows the fertility of its resources, and the attentive industry of the gentlemen concerned in the management of its affairs. The volume now before us, has not only the advantage of early publication, but the materials which compose it are of a class and character that entitles them to considerable approbation.

The first article is by Sir Gilbert Blane, bart. M.D. F.R.S. It consists of "*Facts and Observations respecting Intermittent Fevers, and the Exhalations which occasion them.*"

In the autumn of 1809, Sir Gilbert Blane was sent to the island of Walcheren, for the purpose of ascertaining the nature and causes of the sickness and mortality prevailing in the British army on that island. In the autumn of 1810, he was employed by the admiralty to investigate the local peculiarities of Northfleet in Kent, with a view to decide whether any objection in point of unhealthiness would arise to the formation of a dock-yard, and other naval establishments, at that place.

During Sir Gilbert Blane's visit to those places, the facts detailed in this article were collected.

From the 30th of September to the 13th of October, 1809, this physician was in the island of Walcheren, and during this period he found so great a portion of the sick to consist of those affected with intermitting and remitting fevers peculiar culiar to marshy countries, that no doubt remained that the sickness of the army was owing to that cause.

The history of the Walcheren remittent has been so often detailed, and its morbid phenomena are generally so well known, that instead of any description of the disease, our extracts will be confined to the facts that tend to exemplify the natural history and operation of *marsh miasmata;* an agent still remaining in great obscurity.

In some seasons *marsh miasmata* act with more violence than in others on the same spot. The year of the expedition to Walcheren, it was affirmed by the native inhabitants, was less sickly than usual; and they accounted for this from the unusual quantity of rain that had fallen in July and August; for it is considered by them as a fully established fact, that the most sickly years are those in which there had been great drought and heat in the latter end of the summer, and the early part of the autumn; owing probably to the more concentrated foulness of the stagnant waters. This is perfectly consonant to observations made in the fenny districts of this country. *Marsh miasmata*, when much diluted with aqueous exhalation, is nearly inert; it is when concentrated in the manner just stated, that its power of producing disease is so alarming.

The *miasmata* in Zealand are more noxious than the like exhalations are in England; the intermittents in the former being more violent, untractable, and fatal than those which occur in the fenny countries, in the eastern parts of our own country. Sir G. Blane founds this assertion on the high degree of febrile heat and delirium, on the excessive secretion of bile, on the want of distinct intermissions, and on the more frequent swellings of the liver and spleen, which take place in the Walcheren fever in a few weeks; while in England these (the swellings of the liver and spleen, we suppose, though the structure of the sentence makes the conclusion apply to febrile heat and delirium, &c. as well as to this particular state of the viscera) seldom occur but under a long continuance, or from frequent relapses of the disease.

The distance to which these noxious exhalations, or *marsh miasmata*, extend from their source in a sufficient proportion to produce disease, is a fact so desirable to ascertain, that the observations of Sir G. Blane, which tend to elucidate this circumstance, are peculiarly valuable.

"I had," he says, "in the course of this service, an opportunity of observing the extent to which the noxious exhalations *extended*, which was found to be less than is, I believe, generally known. Not only the crews of ships in the road of Flushing were entirely free free from this endemic, but also the guardships which were stationed in the narrow channel between this island and Beveland. The width of this channel is about 6000 feet; yet, though some of the ships lay much nearer to one shore than to the other, there was no instance of any of the men or officers being taken ill with the same disorder as that with which the troops on-shore were affected. The exhalations from the soil in tropical climates extend farther, and are still more malignant than those of Zealand. Ships at the distance of 3000 feet from swampy shores, (a distance to which it did not extend in Zealand,) and even farther, were affected by the noxious exhalations in the West Indies; and I have been credibly informed of the like fact, with regard to the India ships in the channel which leads to Calcutta."

Another law, governing the diffusion of *marsh miasmata*, seems also to have been ascertained; it is, that neighbouring heights are more certainly affected than flat lands nearer to the source of the *miasmata*, but which flats have not a marshy soil. The following facts go far toward proving this:

"The spot at Northfleet upon which it is intended to erect the dock-yard and arsenal, is a marsh of about 700 acres. On the banks of the river, both above and below it, there is a soil of a similar description, but not immediately adjoining it on either side; for above is the village of Greenhithe, which stands on a chalky bottom, rising to a few inches below the surface, and is a projecting point of the chalky hills which compose the adjacent country. Below it, on the banks of the river, there is a similar intersection of chalk, where the village of Northfleet stands. Both these are nearly on a level with the marsh; yet intermittent fevers are almost unknown at either of them, whereas they are extremely prevalent on the adjacent hills. In the neighbourhood of Weymouth, though there is stagnating water near the sea, producing intermittents, these disorders are not known in the dry districts on each side on a level with the water, but prevail on the adjacent hills. At St. Blazey, between St. Austle and Lestwithiel, agues prevail much on a hill adjoining to a marsh contiguous to the sea beach. In a district on the river Burrampooter, the waters of which overflow, and, upon retiring, leave an oozy flat, agues prevail to the very summit of the adjoining hills. Lancisi mentions a hill on which the same sickness prevails as in the marshy lands at the foot of it. (*Lancisi de Noxiis Paludum Effluviis. Roma,* 1717. p. 120.) At St. Lucia, one regiment, the 90th, on the Morne Fortunée, lost 271 men; the 91st, on the side of the hill, 318; the 89th, in the Grand Cul de Sac at the bottom, 486. The hill or Morne is above the level of the sea 872 feet."

Why this, so contrary to what, *à priori*, might have been expected, occurs, Sir G. Blane endeavors thus to explain:

"It is known to *every* one, *ever* so little acquainted with the operations of nature, and indeed the common phenomenon of clouds and rain render it obvious to the most ordinary observer, that water, re-

 cently cently exhaled from the surface of the earth, has a tendency to ascend, and being lifted over parts on the same level, impinges on the neighbouring heights. There is reason to believe that impure and unwholesome particles in general are attracted by watery vapors; for it is remarkable, that, in case of fogs, offensive smells are perceived, which in a dry state of the air were fixed and quiescent. Though pure humidity, therefore, is innocuous, it may prove pernicious as a vehicle of unwholesome and volatile matter. In like manner, the poisonous principle of marshes, whatever it is, being engendered by moist soils, will naturally adhere to the watery vapors, and ascend with them."

This would go very far to explain why high lands in the neighbourhood of marshes are so commonly enveloped in a poisonous dose of the *miasmata*, did it not occur, as has been previously asserted, that these *miasmata* are in the most active state during dry and hot autumns. It remains, however, matter for future observation, and we hope it will not be lost sight of, whether the *miasmata* become more active after a thunder storm, sometimes occurring in a series of dry weather, or on the setting in of wet; or whether they are really more active in a *dry* than in a *humid* atmosphere. The fact of marshy effluvia being most pernicious in dry and hot autumns we consider to be fairly established; but we are not aware that a humid atmosphere is at all essential to their rapid and destructive diffusion.

Did our limits permit, we could add with much satisfaction many more extracts from this valuable paper; and, though we cannot compliment the baronet on the elegance or perspicuity of his style, we acknowledge the value of his materials. An appendix to this paper, containing "Remarks on the comparative Health and Population of England and Wales," though it goes but slightly into the subject, contains some interesting facts, which we may hereafter take occasion to present to our readers.

### Art. II. *History of a remarkable Case of Ovarian Dropsy. By* Thomas Chevalier, Esq. F.L.S.

This occurred to a woman 23 years of age; it was of six years standing when Mr. C—— first saw it: the abdomen enormously large, but unaccompanied with any alteration of the general health, except that the catamenial discharge had failed for two years, and there was a scanty secretion of urine. The circumference of the abdomen was 63 inches and a half; 38 inches from the point of the ensiform cartilage to the top of the pubis. The legs were œdematous, and a great part of the skin of the left leg was in a complete ichthyosis. The lower part of the belly was also œdematous, and the navel when she sat was on a line with the knee.

knee. On the 1st of June an oblique puncture with a lancet was made at the most prominent part of the abdomen, in the linea alba, six inches above the navel, and seven gallons and a half of coffee-colored fluid were evacuated. On the 8th a second puncture was made in the most prominent part on the left side, and four gallons and a half of a straw-colored ropy fluid were discharged. A puncture with a trocar was made on the left side on the 27th, and a gallon and a half of a very ropy brownish fluid was discharged. July the 6th a small quantity of fluid was evacuated by the trocar, but a cyst of uncommon firmness closed the canula; this cyst was punctured with the trocar used for puncturing the bladder through the rectum, and three gallons and a half of ropy brown fluid were drawn off, completely emptying the abdomen.

After this last operation her health became much disordered, and she languished till the 6th of September, when she died.

On examining the body after death, the whole cavity of the abdomen appeared to be occupied by two large cysts formed in the left ovarium, of a firm texture. The upper of these cysts contained about two quarts of a brown glary fluid, the inferior about two gallons of purulent fluid; its inner surface was covered with coagulable lymph, having in it many large dark-red spots. The right ovarium and uterus were in a perfectly healthy state.

The interesting particulars of this case were, the enormous quantity of fluid accumulated in these cysts, not less than seventeen gallons at one time; the little derangement in the health from extraordinary accumulation, almost the whole of the inconveniences she sustained arising from the excessive weight; and the inflammation and suppuration in the larger and inferior cyst, to which her death is to be ascribed.

It is a curious pathological fact, that in this cyst, "near a quarter of an inch in thickness, as compact in its texture as parchment, and entirely insensible, the process of inflammation should be excited, and go on to so large a collection of pus, attended with a rapid decay of the health, and showing the sympathy of the constitution with this adventitious substance, without pain or tenderness being excited in it, or the neighbouring parts."

Art. III. *A Case of difficult Parturition, occasioned by a Dropsical Ovarium forming a Tumor in the lower Part of the Pelvis. By* Samuel Merriman, M.D. &c.

Dr. Merriman was called to a woman in labour with her second child; the process of parturition seemed to be retarded

 by by a tumor in the pelvis. On examination *per vaginam*, this tumor was ascertained to be soft and elastic, seemed capable of containing four or five ounces of fluid, was compressible, did not give the sensation of fluctuation, but rather felt as if it were a large pouch, formed by the coats of the rectum preternaturally distended. Beyond this tumor the child's head was felt within the os uteri, which was thick, rigid, and but little dilated. After thirty-six hours of ineffectual labour pains, it was deemed proper to have recourse to the perforator. After diminishing the size of the head, the child was expelled; and a second of smaller dimensions by the natural pains.

On the twenty-fifth day after delivery, the woman died of peritoneal inflammation.

"The body was opened the next day, and exhibited the usual appearances of peritoneal inflammation. The left ovarium was in its proper situation, and of its usual size; but the right was found lying between the rectum and vagina, and had formed the tumor felt during labour. It was about the size of a small trap-ball, and in a state of high inflammation. It was divided into several cavities by membranous *septa*, which had a scirrhous feel, and was somewhat more than half filled with a fluid resembling in color and consistence honey and water mixed together; it contained likewise a clot of blood. The pelvis was narrow, the diameter from the symphisis pubis to the sacrum being but little more than three inches."

The ingenious author observes on this, "that though the narrowness of the pelvis would have delayed the passage of the head through the upper aperture, it is not improbable that the presence of twins might likewise prevent the full effect of the pains." But he imagines that the position of the diseased ovarium, more than any thing else, prevented the expulsive efforts of the womb; not because it proved an obstacle to the birth of the child, but by paralysing all regular uterine action.

Several instances are adduced from authors, of tumors in the pelvis retarding the process of parturition.

ART. IV. *Case of diseased Appendix Vermiformis. By* JOHN PARKINSON, Esq. Surgeon.

Inflammation and ulceration occurred, and through an opening in the appendix a fetid fluid escaped into the cavity of the abdomen.

ART. V. *A Case of Diseased Testicle, accompanied with Disease of the Lungs and Brain, and terminating fatally. By* HENRY EARL, Esq. *To which is added a Note, by* WILLIAM LAWRENCE, Esq. *containing some Particulars of the Histories and Dissections of four Cases.*

A boy, one year and nine months old, had, when a year old, old, received a pinch on the testicle. From that period the gland gradually increased. He had been under the care of several surgeons; and leeches, poultices, mercurial ointment, &c. had been employed without producing any visible amendment. The case had been twice mistaken for hydrocele, and the testicle had been punctured with a trocar, but no fluid was evacuated. But little inflammation succeeded these operations, and the disease did not appear to be aggravated by them. At this period the testicle was rather larger than a goose-egg, and, when unsupported, reached to the internal condyle of the fenur. It was of an oval figure, with a regular smooth surface, and when pressed had an elastic feel, so as to produce the sensation of a fluid contained in a cyst; and so deceptive was this feel, that a gentleman, who has most extensive practice in the treatment of hydrocele, pronounced it to be that disease. It was not, however, in the slightest degree diaphanous; and, at the same time, was much heavier than a similar bulk of water. No testicle or epididymis at the posterior or inferior part of the tumor. The child had a most unhealthy aspect; the skin a greenish yellow, bedewed with a clammy moisture; the muscles flaccid, and diminished in size; head large, and prominent in front; eyes heavy; pupil dilated; and the iris of so unusually dark a color, as to be hardly distinguished from the pupil. Respiration rather laborious, troublesome cough, with frequent and hard pulse; abdomen tense, and habitually constipated.

The testicle was removed, the child regained health, and appearances were favorable for some months; but a disease in the brain and lungs, connected, as it does seem, with a peculiar morbid diathesis, which exists with this disease of the testicle, destroyed him. The dissection, previous history of the disease, and the subsequent remarks of Mr. Lawrence, illustrated by four additional cases, give considerable interest to this paper.

ART. VI. *Description of an improved Method of tying diseased Tonsils.* *By* THOMAS CHEVALIER, Esq.

This method of passing a ligature round a diseased tonsil, cannot be made intelligible to our readers without the accompanying plate.

ART. VII. *Case of Cynanche Laryngea.* *By* J. R. FARRE, M. D.

Something like novelty which attaches to cases of *Cynanche Laryngea*, the celebrity of some men who have fallen by it, and its fatal celerity, combine to give it importance. The two cases by Dr. Farre are of great interest, though related related with much conciseness. The second of them, as having come immediately under his notice, we shall give in the author's words.

"A man, 60 years old, on the 31st of March was affected with painful and difficult deglutition. April 1st, fluids attempted to be swallowed returned by nose; the tonsils were inflamed, and disposed to ulcerate. A brisk purge was ordered. At ten o'clock this evening, his respiration suddenly became difficult; thirty-two ounces of blood was drawn from his arm, which proved to be very sizy. At eleven o'clock, the tumefaction of the tonsils was inconsiderable; the deglutition was extremely painful, and very difficult; respiration was performed with convulsive and long-continued efforts; his voice was nearly inaudible, and he could only whisper. He answered inquiries respecting the seat of his suffering, by putting his finger on the superior part of the thyroid cartilage. He felt no pain in the chest. All the muscles of respiration were thrown into violent action, and he lay with his mouth widely opened, pupils dilated, face pale and sunken, skin covered with a clammy sweat. his pulse 133 and small. His powers were prostrate, and general bleeding could not be repeated. The anterior part of his throat was covered with leeches, but the disease never paused. At midnight, bronchotomy seemed to be the only resource; and soon after one o'clock its employment was decided on. About two o'clock Mr. Astley Cooper attended, and as suffocation was instantly impending, the operation was immediately performed, by dividing, laterally, the ligament which connects the thyroid with the cricoid cartilage. The dyspnœa was much relieved by the operation. He now lay passive, breathing by the natural and artificial aperture, and the inordinate action of the muscles of respiration had ceased. He swallowed some nourishment with a painful effort. In this state he passed the night, and the greater part of the following day. In the afternoon, the respiration by the natural passage entirely ceased, but was continued by the artificial aperture. He was now evidently sinking, and expired at six in the evening."

At eight o'clock the next morning, the parts, as examined by Mr. Astley Cooper, gave the following appearances:

"The right tonsil inflamed and vesicated. The epiglottis swollen, its edges meeting behind, excepting just at its upper part. Pharynx inflamed, somewhat vesicated, covered with coagulable lymph about the epiglottis, but free from inflammation near its termination in the œsophagus. The aperture which had been made between the cartilages, appeared to be about the size of the glottis. The mucus membrane of the larynx and trachea was pale. There was some accumulation of mucus in the cells of the lungs, and a slight affusion of serum into their reticular texture."

The term *Cynanche Laryngea* is properly applied to this disease, Dr. Farre observes, because it proves fatal by constricting or actually closing the glottis, and constitutes precisely that case which, in its ultimate degree, imperiously demands the operation of *bronchotomy.*

ART.

ART. VIII. *History of a Case of Anæthesia. By* JOHN YELLOLY, M.D.

This is a correctly written history of an unusual disease, and, as a collection of facts of a nature so singular as to confound some of our physiological opinions on the action of the brain and nerves, deserves the serious attention of those who endeavor to explain the actions of those organs. The loss of sensation in the upper and lower extremities, independent of paralysis, at the same time that the integuments of these parts were more readily acted upon by certain external agents, (particularly vesication was produced by exposure to a degree of heat quite unequal to the production of it in a healthy skin) involve a contradiction not to be explained by any existing systems. Our limits do not permit an insertion of the case. Dr. Yelloly has annexed a collection of similar instances of Anæthesia found in authors.

ART. IX. *Account of a Case of spontaneous Extravasation within the Theca Vertebralis. By* THOMAS CHEVALIER, Esq.

This paper contains a short history of instances of extravasation of blood into the cavity of the spinal canal, constituting a species of apoplexy in which the brain is not directly concerned. It is conjectured to be produced by some violence *ob extra*, and is often rapidly fatal. Bleeding, and the antiphlogistic treatment very early employed, seems to afford the most rational means of obviating the impending danger.

ART. X. *Observations on Diabetes Insipidus. By* JOHN BOSTOCK, M.D.

*Diabetes insipidus* is considered, by Dr. Bostock, as of rare occurrence;* and he believes this to be very decidedly proved by the fact of scarcely any distinctly detailed case of this disease being recorded since the time of Willis, who first pointed out the sweetness of the urine. Cullen says (First Lines, 4, 85) that he had seen but one case in which the urine was not saccharine. The two cases related by Dr. Rollo, in which a large quantity of watery urine was evacuated, were consequent to a local injury of the kidney, and do not belong to this class. The case in Duncan's Annals (1801, p. 390—1802, p. 361) is not entitled to the

* In the course of his remarks, however, Dr. Bostock seems, in no small degree, to have given up this opinion. At p. 114, he says, "for although cases of *Diabetes insipidus* have been but seldom noticed, yet I am disposed to believe that it is a more frequent occurrence than is generally imagined."

appellation of diabetes. The only reference made by Cullen is to Lister; but it appears that Lister believed in the existence of *Diabetes insipidus* merely upon hypothetical grounds. Sauvages hastily and peremptorily declares that all the diabetic cases of the ancients were insipid, because they do not mention the sweetness of the urine; while it is admitted that ever since the time of Willis, all the cases have been saccharine. (Nos. Meth. 2, 384.) It may not be improper to observe, that the opinion that the stomach and not the kidney was the primary seat of diabetes, is not a novelty, for Lister (Exerc. Med. p. 74) implicitly declared and defended this opinion a century ago.

The case related by Dr. Bostock was cured by the use of preparations of iron and the warm bath. The greater part of this paper is occupied by chemical experiments with this urine, and that of other diabetics.

Art. XI. *Cases of premature Labour artificially induced, in Women with distorted Pelvis; to which is subjoined, some Observations on this Method of Practice. By* Samuel Merriman, M.D.

The whole routine of medical practice does not present any other cases which require so great a share of delicacy and caution, as those demanding an artificial inducing of premature labour. The subject has been treated with, perhaps, a proper reserve; which has, consequently, left the practice too little explained. Under these circumstances, the profession must be much indebted to this ingenious and able physician for going thus fully into the subject. No analysis will do justice to this article, and we unreservedly recommend it to the attention of our readers.

Art. XII. *Experiments on the Bark of the Cocoloba Uvifera. By* John Bostock, M.D.

The *Cocoloba Uvifera*, or Mangrove grape tree, belongs to a genus of which many species are known. It is common in most of the sugar colonies, and is generally found near the sea. By the Spaniards it is called *Uvero*, and by the French *Raisenier du bord de la Mer*. It was brought into this country by Mr. Bentick in 1696.

Dr. Bostock has been employed on an examination of the chemical properties of the bark of this species of *Cocoloba*, with a reference to its identity with the *Kino* of the West Indies: the analysis is here laid before the public, and the result is that the extract of *Cocoloba* is a substance of the same nature with the *Kino* used in medicine; but that it differs from it so far as to show that they are not derived from the same plant.

Art.

**Art. XIII.** *A Case of Splenitis, with further Remarks on that Disease. By* Robert Bree, M.D. F.R.S.

This is a case of great importance in practice, presenting a detail of the phenomena of a complaint of much obscurity, and liable to very improper treatment, if not absolutely to be mistaken or neglected. It is intended to show the advantages of a course of drastic purging in *Splenitis*. The extensive employments of the author have brought under his view numerous cases of this disease; and his talent for observation has enabled him to detect circumstances, and direct methods of treatment, which have not occurred to less attentive practitioners.

"Many cases," he says, "have concurred to show me in a very satisfactory manner that drastic purging, long continued, is the proper mode of treatment. By this practice a young woman has been relieved of a swelling of the spleen and epileptic fits at the same time. The fits began with the first symptoms of disease in the left side, and have disappeared for the last year, during which time she has been gradually recovering from the swelling and pain. Compositions of aloes and antimony were preferred in the cases that have been related, but not exclusively adopted: large doses of neutral salts have, however, appeared exceptionable when exhibited daily, as they have occasioned flatulence and depression. But aloes, extract of colocynth, and scammony with jalap, have acted without this inconvenience, and calomel has been combined with these at intervals, producing more effectual discharges from the bowels: tartarized antimony in such minute doses as not to puke, has always appeared to increase the beneficial effect of these combinations."

Of the progress of *Splenitis* in its different stages, as marked by symptoms, the description seems so distinct and specific, that we cannot refuse to lay it before our readers, as affording that valuable kind of information directly applicable to practice.

"In the earliest condition of this disorder, the organ is swelled from the passive state of its vessels, which receive a greater proportion of blood than they can return. No fever accompanied this stage, nor was it the effect of fever, but an idiopathic affection, leading to inflammation by tension and irritation of the membranes that invest the spleen. The means of cure were experienced to be active, and daily so persisted in as to become the probable cause of disease, if they had not been essential to the removal of it.

"In the second stage the pulse becomes quicker, and it is long in convalescence before it is reduced to its natural standard. The increased pulse is produced by painful irritation at first, and next by the actual tension of the membranes, proceeding to inflammation and adhesion of the adjoining parts. The quickness of the pulse will assist in distinguishing the degree of progress of this disease, for it will be found, by reference to histories, that in a great proportion of cases, there was no warning of the growing mischief in the earliest stage; and that painful affection of the left side existed in many other cases, long before fever was induced, though these ended fatally. In the first stage the patient can lie upon the left side, but not upon the right side. In the second stage it is impossible to lie on the side affected. The spasmodic action of the diaphragm is more likely to come on in the second stage, and may be much aggravated by stimulant treatment. There is no emaciation in the first stage of a morbid kind, nor any considerable emaciation in the second stage, notwithstanding the large and continued evacuations.

"In the third and last stage of *Splenitis*, emaciation is always an attending symptom, combined with hectic or slow fever, particularly in middle-aged and elderly people. In this third stage diarrhœa supervenes, as well as dysentery, and discharges of grumous and dark blood take place, by vomiting and by stools: these discharges give temporary relief in many cases, and occur long before the final event."

Art. XIV. *Account of the Muscles of the Ureters, and their Effects in the irritable State of the Bladder. By* Charles Bell, Esq. F.R.S. Ed.

A description of a set of muscles, which Mr. Bell considers have not been noticed by former anatomists. They are attached to the orifices of the ureters, and are seated in the bladder. In health they are the instruments of a very peculiar organic action, and in disease the cause of most distressing complaints. Among other interesting details, the writer goes into an historical inquiry concerning the existence of a third lobe of the prostate, and shows that it was known to *Morgagni*, and had become the subject of discussion in his day. Plates and diagrams illustrative of the author's positions and descriptions, accompany this paper.

Art. XV. *History of a Case in which a Calculus was voided from a Tumor in the Groin. By* Thomas Copeland, Esq.

This well-written account and judicious management of an extraordinary case, deserves the attention of the profession, and is a very positive instance of the powers of the *Vis Medicatrix* when not obstructed by the improper interference of art. In the case, where the calculus was discharged at the abscess in the groin, it is understood to have been lodged in the *cæcum*.

(To be continued.)

MEDICAL

